# High baseline CD317 expression on T cells predicts favorable anifrolumab response in systemic lupus erythematosus

**DOI:** 10.3389/fimmu.2026.1756139

**Published:** 2026-03-26

**Authors:** Koichi Kimura, Masahiro Ayano, Shun-Ichiro Ota, Kazuo Kushimoto, Yasushi Inoue, Atsushi Tanaka, Keisuke Imabayashi, Sho Fujimoto, Naoya Nishimura, Ayako Takaki-Kuwahara, Yasutaka Kimoto, Hiroki Mitoma, Nobuyuki Ono, Koichi Akashi, Takahiko Horiuchi, Hiroaki Niiro

**Affiliations:** 1 Department of Medicine and Biosystemic Science, Kyushu University Graduate School of Medical Sciences, Fukuoka, Japan; 2 Department of Rheumatology, Shimonoseki City Hospital, Shimonoseki, Japan; 3 Department of Rheumatology, Fukuoka Red Cross Hospital, Fukuoka, Japan; 4 Department of Medical Education, Kyushu University Graduate School of Medical Sciences, Fukuoka, Japan; 5 Department of Internal Medicine, Kyushu University Beppu Hospital, Beppu, Japan; 6 Department of Rheumatology, Fukuoka City Hospital, Fukuoka, Japan

**Keywords:** anifrolumab, biomarkers, flow cytometry, precision medicine, systemic lupus erythematosus, type I interferon

## Abstract

**Background:**

Anifrolumab (ANI), a monoclonal antibody blocking the type I interferon (IFN) receptor, is approved for systemic lupus erythematosus (SLE); yet real-world responses vary. We aimed to identify biomarkers predicting clinical response to ANI in SLE.

**Methods:**

We prospectively enrolled patients with SLE who initiated ANI and evaluated immune cell subsets and type I IFN-associated markers by flow cytometry with respect to clinical response to ANI. Clinical response at 6 months was classified into responders and non-responders based on two criteria: achievement of a British Isles Lupus Assessment Group-based Composite Lupus Assessment (BICLA) response and successful glucocorticoid tapering.

**Results:**

Of the 31 patients analyzed, 15 were responders and 16 were non-responders. Among the ten biomarkers that showed significant changes in responders, four - CD317 expression on T cells, CD317 expression on B cells, CD169 expression on monocytes, and T-peripheral-helper–cell frequency - were already higher at baseline in responders than in non-responders. Baseline CD317 expression on T cells showed the highest discriminative power in predicting 6-month response, separating responders from non-responders with an AUC of 0.89, surpassing the four-gene IFN gene signature (IFNGS) measured by quantitative PCR (qPCR) (DeLong’s test, *P* = 0.044).

**Conclusions:**

This study demonstrates that higher baseline CD317 expression on T cells is associated with a favorable clinical response to ANI and predicts this response more accurately than the previously proposed IFNGS in patients with SLE. These findings identify CD317 as a promising and practical candidate biomarker to guide personalized treatment strategies in SLE, contingent upon further validation.

## 1 Introduction

Systemic lupus erythematosus (SLE) is a chronic systemic autoimmune disease characterized by marked heterogeneity in clinical manifestations, prognosis, and immune dysregulation. Therefore, precision medicine approaches that tailor treatment to individual pathophysiological profiles are needed (1–4). High expression of type I interferon (IFN)-stimulated genes is well demonstrated in peripheral blood and affected tissues of patients with SLE, thereby highlighting the pivotal role of type I IFN signaling in disease pathogenesis (5–7).

Anifrolumab (ANI), a human monoclonal antibody that blocks the type I IFN receptor, reduced overall disease activity and enabled sustained glucocorticoid tapering in the phase III TULIP-1 and TULIP-2 trials, leading to its recent approval for the treatment for SLE (8, 9). In real-world practice, however, the clinical response to ANI is heterogeneous; some patients benefit substantially, whereas others do not. Consequently, there is a strong need to identify baseline predictors of treatment response. Mechanistically, patients with excessive type I IFN signaling would be expected to benefit more from receptor blockade. Indeed, a *post hoc* analysis of the phase III TULIP-1 and TULIP-2 trials demonstrated greater efficacy in patients with an elevated interferon gene signature (IFNGS) (10). Even so, not all patients with high IFNGS respond uniformly (8–10). One possible explanation is that IFNGS can also be induced by type II or type III IFNs and therefore does not necessarily reflect specific type I IFN activity (11). Gene expression analyses have shown that type I and type III IFNs induce an almost identical set of genes, with no gene uniquely upregulated by type III IFNs (12), although the tissue distribution of their receptors differs substantially. IFNGS by type I and type II IFNs may overlap as well (13). Moreover, circulating levels of type I IFN do not always correlate with its functional activity (11, 14), and methodological and standardization issues make it difficult to quantify type I IFN signaling accurately in routine clinical settings.

As a complementary approach, flow cytometry-measurable cell surface markers have attracted increasing attention. CD317 (BST2) and CD169 (SIGLEC-1) are upregulated almost predominantly by type I IFNs (IFN-α/β), whereas stimulation with type II (IFN-γ) or type III (IFN-λ) IFNs results in only minimal changes in their expression (15, 16). Because the expression of these markers reflects increased type I IFN activity, they may serve as potential predictors of response to ANI. Moreover, as type I IFN signaling is thought to modulate a wide range of immune phenotypes (17), capturing such alterations could provide additional insights into mechanisms underlying variable treatment response.

To identify novel biomarkers predictive of treatment response in SLE, we performed a comprehensive longitudinal analysis of clinical parameters and immune cell subsets before and after ANI initiation. Specifically, we evaluated a broad spectrum of immunologic parameters - including absolute cell counts, fine-grained subset composition across the B-, T-, monocyte-, dendritic-, and NK-cell lineages, and the expression of selected type I IFN-inducible surface markers (e.g., CD317, CD169) - to determine whether any of these baseline features were associated with subsequent response to ANI.

## 2 Materials and methods

### 2.1 Patients

This study was conducted within the Kyushu Collagen Disease Network for SLE (KCDN-SLE) registry, an ongoing multicenter prospective cohort registry. We enrolled patients with SLE who initiated ANI between August 2022 and March 2025 at three sites where biospecimens were accessible - Kyushu University Hospital, Japanese Red Cross Fukuoka Hospital, and Shimonoseki City Hospital. Patients who fulfilled the 2019 ACR/EULAR classification criteria for SLE (18) and were aged 20 years or older were included. Patients who discontinued ANI before the second infusion were excluded from all analyses. All included patients received ANI 300 mg intravenously every four weeks in addition to standard treatment. Adjustments in immunosuppressants or glucocorticoids (GCs) were permitted at the discretion of the attending physician. The study was approved by the Ethics Committee of Kyushu University Hospital (approval No. 22041-01) and conducted in accordance with the Declaration of Helsinki. Written informed consent was obtained from all participants.

### 2.2 Clinical and laboratory assessment

Patients were evaluated at baseline (before the first ANI infusion) and 1, 3, and 6 months after ANI initiation. At each visit, peripheral blood was collected into heparinized tubes. PBMCs were isolated by density-gradient centrifugation, resuspended in Cell Banker 1 cryopreservation solution (Zenogen Pharma Co., Ltd., Koriyama, Fukushima, Japan), and stored at -80°C until flow-cytometric or quantitative real-time PCR analysis. Concomitant laboratory tests included complete blood counts, serum chemistry, complement levels, anti-double-stranded DNA antibodies (anti-dsDNA), and urinalysis. Demographic data, clinical characteristics, disease activity, organ involvement, and medications were extracted from patients’ medical records. Disease activity and organ involvement were assessed at every visit using the SLE Disease Activity Index 2000 (SLEDAI-2K) (19) and the British Isles Lupus Assessment Group 2004 index (BILAG-2004) (20). Changes in glucocorticoid (GC) dosage and concomitant immunosuppressant (IS) use were documented. Reasons for treatment discontinuation after two or more infusions (adverse event, inadequate efficacy, other reasons) were also recorded.

### 2.3 Outcomes

The primary outcomes were achievement of a BILAG-based Combined Lupus Assessment (BICLA (21);) response and successful GC dose reduction. For patients who exhibited at least one BILAG grade A or B involvement at baseline, a responder was defined as meeting the BICLA criteria at month 6, consistent with the primary endpoint of the pivotal TULIP-2 trial. In contrast, for patients without BILAG grade A or B involvement whose main treatment objective was GC reduction - a common goal in routine clinical care -, a responder was defined as (i) having no flare recorded by the BILAG-based Flare Index (BBFI) (9, 22) during the 6-month observation period and (ii) achieving a ≥ 25% reduction in GC dose from baseline. All other patients were classified as non-responders. To assess robustness across response definitions, a sensitivity analysis was conducted using an alternative responder definition. A responder was defined as meeting either of the following criteria without treatment intensification or flare: (i) a ≥1-point reduction in SLEDAI-2K score from baseline, or (ii) a ≥25% reduction in GC dose from baseline.

### 2.4 FACS analysis

Peripheral-blood mononuclear cells (PBMCs) collected at baseline (month 0) and at months 1, 3, and 6 were thawed and stained on the same day in a single flow cytometry batch using the anti-human monoclonal antibodies listed in **Supplementary Table 1**. Isotype control antibodies (BD Biosciences) were used to determine background staining. Data were acquired on a FACSAria II flow cytometer (BD Biosciences, Franklin Lakes, NJ, USA) and analyzed with FlowJo software version 10.8.0 (BD Biosciences). Absolute counts (cells/µL) of major lymphocyte subsets - T cells and B cells - and monocytes were calculated by combining complete blood count data with flow cytometric gating frequencies. Subsets forming discrete populations (e.g., naïve or memory B cells) were expressed as percentages, whereas markers exhibiting a continuous distribution within a subset (e.g., CD317 on B cells or CD169 on monocytes) were quantified by median fluorescence intensity (MFI). Representative gating plots and CD317 histograms are shown in **Supplementary Figure 1**. MFI values were obtained using the “Median” output and are reported herein as “MFI.”

Subset definitions followed the NIH/Federation of Clinical Immunology Societies Human Immunology Project protocols (23); subsets or markers not included in the core NIH protocol were added according to published gating strategies as follows: type I IFN–responsive markers - CD317 (BST2) and CD169 (SIGLEC1); B-cell subsets - double-negative 2 B cells (DN2; CD11c^+^ CXCR5^-^ IgD^-^ CD27^-^) (24), BAFF-R^+^, TACI^+^, BCMA^+^, and Tim-1^+^ regulatory B cells (Bregs) (25); T-cell subsets - age-associated T helper cells (ThA; CXCR3mid CD4^+^ Tem) (26) and peripheral helper T cells (Tph; PD-1hi CXCR5^-^) (27).

### 2.5 Biomarker analysis

Fold change for each marker was calculated as the month-6 value divided by the baseline value, log_2_-transformed to approximate normality, and analyzed. Samples lacking the relevant subset at baseline or missing month-6 data were excluded from the biomarker analysis. Markers that changed significantly within responders were shortlisted; and those whose baseline levels also differed between responders and non-responders were subsequently subjected to receiver operating characteristic (ROC) analysis.

### 2.6 Quantitative real-time PCR

Total RNA was isolated from baseline PBMCs using the RNeasy Micro Kit (QIAGEN, Hilden, Germany) and reverse-transcribed with ReverTra Ace qPCR RT Master Mix (TOYOBO, Osaka, Japan). To comprehensively evaluate type I IFN signaling, transcript levels of the six most frequently reported ISGs—MX1, IFI44L, IFIT1, IFI44, RSAD2, and IFI27—were selected based on a recent EULAR systematic literature review of type I IFN pathway assays (28). These transcripts were quantified in triplicate by TaqMan assays on 96-well plates with a CFX Connect Real-Time PCR Detection System (Bio-Rad, Hercules, CA, USA). Assay IDs are provided in **Supplementary Table 2**. Expression of 18S rRNA served as the endogenous control. Cycle-threshold (Ct) values were exported with CFX Maestro software, normalized to 18S rRNA (ΔCt), and expressed relative to healthy controls using the 2^ - ΔΔCt method. Values were log_2_-transformed, and the arithmetic mean of log_2_-transformed IFI27, IFI44, IFI44L, and RSAD2 values were defined as the four-gene interferon signature (4GS) (9, 28). Using the same approach, the arithmetic mean of log_2_-transformed MX1, IFI44L, IFIT1, IFI44, RSAD2, and IFI27 values was defined as the six-gene interferon signature (6GS).

### 2.7 Biomarker-based prediction (ROC curves)

Baseline values of the final candidate biomarkers - that is, markers that both changed significantly within responders and differed at baseline between responders and non-responders in the preceding screening step - together with the 4GS were subjected to ROC analysis to evaluate their ability to discriminate responders from non-responders at month 6. As a sensitivity analysis, ROC analyses were also performed for the 6GS and for each of the six ISG transcripts (MX1, IFI44L, IFIT1, IFI44, RSAD2, and IFI27).

### 2.8 Correlation analysis

To explore clinical relevance, we first examined correlations between final candidate biomarkers and continuous clinical variables - SLEDAI score, anti-dsDNA antibody titer, GC dose, complement C3/C4 levels, and the 4GS - using Spearman’s rank correlation. We then compared biomarker levels across categorical variables; autoantibody status (anti-Sm, anti-RNP, anti-SSA/Ro); presence of BILAG grade A or B involvement (A1/B1); major organ involvement (mucocutaneous, musculoskeletal, hematologic, or renal); concomitant medications at ANI initiation (hydroxychloroquine [HCQ] or other immunosuppressants); and previous belimumab (BLM) treatment.

### 2.9 Statistical analysis

Continuous variables are presented as mean ± standard deviation or as median with an interquartile range (IQR), as appropriate; categorical variables are reported as frequencies and percentages. Group comparisons were performed using the Student’s *t* test for normally distributed continuous variables, the Mann-Whitney *U* test for non-normally distributed continuous variables, and the Fisher’s exact test or the chi-square test for categorical variables. Within-group changes in biomarkers from baseline to month 6 for biomarkers were evaluated using two-tailed one-sample Student’s *t* tests on log_2_-transformed fold changes. *P*-values were adjusted for multiple testing with the Benjamini–Hochberg false discovery rate (FDR) procedure. Correlations between continuous variables were assessed using Spearman’s rank correlation. ROC analysis was used to estimate the area under the curve (AUC) with 95% confidence intervals. Bootstrap-based internal validation (2,000 iterations) with out-of-bag evaluation was conducted to estimate an optimism-corrected AUC. Optimal cutoffs were defined by maximizing Youden’s *J* index, and the corresponding sensitivity and specificity were reported. All statistical tests were two-sided, and significance was defined as *P* < 0.05 (for single comparisons) or adjusted *P* < 0.05 after FDR correction. All analyses were exploratory, and no formal sample size calculation was performed. Statistical analysis were conducted using GraphPad Prism 9 (GraphPad Software, San Diego, CA, USA) and R (R Foundation for Statistical Computing, Vienna, Austria).

## 3 Results

### 3.1 Patient characteristics

**Figure 1:**
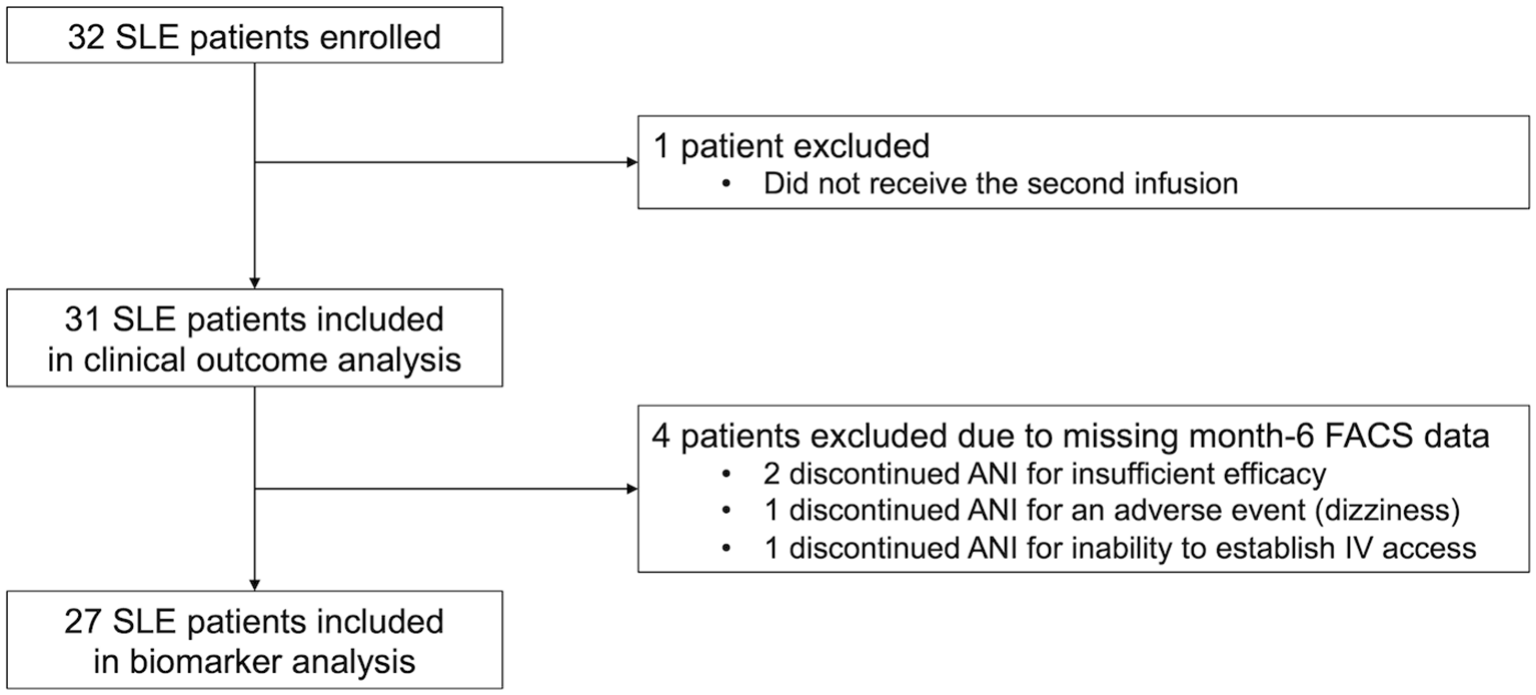
Algorithm for the inclusion and exclusion of the study population.

The study enrolled 32 patients with SLE who initiated ANI during the observation period. One patient discontinued ANI after the first infusion and was excluded, leaving 31 patients for analysis (**Figure 1**). The mean ± SD age was 39.9 ± 15.0 years, all patients were female, and the median disease duration was 10 (IQR 5.5–17.5) years (**Table 1**). At baseline, disease activity was generally low to moderate. The median SLEDAI was 4.0 (IQR 2.0 - 6.0). Overall, 68% of patients had grade A or B involvement in at least one domain, and 22% had at least one BILAG grade A domain. Mucocutaneous involvement was most common, followed by musculoskeletal and hematologic involvement. At baseline, the median prednisolone (PSL) dose was 5.0 mg/day (IQR 4.0-9.5). Concomitant medication use was frequent: approximately 60% received an IS, and 35% had prior BLM treatment.

**Table 1:** Baseline clinical characteristics of patients with SLE.

Variable	All (n=31)	Responders (n=15)	Non-responders (n=16)	P-value
Age, years, mean ± SD	39.9 ± 15.0	35.9 ± 12.5	43.7 ± 16.6	0.15
Female, n (%)	31 (100.0%)	15 (100.0%)	16 (100.0%)	–
Disease duration, years	10.0 [5.5-17.5]	7.0 [4.0-15.5]	11.5 [8.0-21.0]	0.14
SLEDAI score	4.0 [2.0-6.0]	4.0 [2.0-6.0]	4.0 [2.0-6.0]	1
Serological activity, n (%)	15 (48.4%)	5 (33.3%)	10 (62.5%)	0.16
Low C3 and/or C4, n (%)	8 (25.8%)	2 (13.3%)	6 (37.5%)	0.22
C3, mg/dL	92.0 [73.5-105.0]	100.0 [77.0-117.0]	89.0 [64.2-101.8]	0.21
C4, mg/dL	16.0 [11.5-21.0]	16.0 [14.5-21.5]	13.0 [10.2-21.0]	0.33
Anti-dsDNA antibody positivity, n (%)	11 (35.5%)	4 (26.7%)	7 (43.8%)	0.46
Anti-Sm antibody positivity, n (%)	6 (19.4%)	3 (20.0%)	3 (18.8%)	1
Anti-RNP antibody positivity, n (%)	15 (48.4%)	6 (40.0%)	9 (56.2%)	0.48
Anti-SSA/Ro antibody positivity, n (%)	16 (51.6%)	9 (60.0%)	7 (43.8%)	0.48
Organ manifestations, n (%)				
Mucocutaneous - BILAG grade A-C	20 (64.5%); A/B/C: 6/8/6	11 (73.3%); A/B/C: 4/4/3	9 (56.3%); A/B/C: 2/4/3	0.46
Musculoskeletal - BILAG grade A-C	11 (35.5%); A/B/C: 0/4/7	7 (46.7%); A/B/C: 0/3/4	4 (25.0%); A/B/C: 0/1/3	0.27
Renal - BILAG grade A-C	2 (6.5%); A/B/C: 0/2/0	0 (0.0%); A/B/C: 0/0/0	2 (12.5%); A/B/C: 0/2/0	0.48
Hematologic - BILAG grade A-C	16 (51.6%); A/B/C: 1/1/14	6 (40.0%); A/B/C: 0/0/6	10 (62.5%); A/B/C: 1/1/8	0.29
Disease status				
BILAG A ≥ 1	7 (22.6%)	4 (26.7%)	3 (18.8%)	0.69
BILAG A or B ≥ 1 (A1/B1)	21 (67.7%)	11 (73.3%)	10 (62.5%)	0.7
Concomitant medications at baseline				
PSL dose, mg/day	5.0 [4.0-9.5]	5.0 [3.5-15.0]	5.0 [4.0-8.2]	0.59
PSL > 7.5 mg/day, n (%)	10 (32.3%)	5 (33.3%)	5 (31.2%)	1
HCQ	25 (80.6%)	12 (80.0%)	13 (81.2%)	1
IS	19 (61.3%)	9 (60.0%)	10 (62.5%)	1
Antimetabolite use, n (%)	10 (32.3%)	4 (26.7%)	6 (37.5%)	0.7
MMF	6 (19.4%)	1 (6.7%)	5 (31.2%)	0.17
MZR	3 (9.7%)	2 (13.3%)	1 (6.2%)	0.6
AZA	0 (0.0%)	0 (0.0%)	0 (0.0%)	–
MTX	1 (3.2%)	1 (6.7%)	0 (0.0%)	0.48
Calcineurin inhibitor use, n (%)	16 (51.6%)	7 (46.7%)	9 (56.2%)	0.72
TAC	11 (35.5%)	5 (33.3%)	6 (37.5%)	1
CsA	5 (16.1%)	2 (13.3%)	3 (18.8%)	1
Combination with antimetabolite and calcineurin inhibitor, n (%)	7 (22.6%)	2 (13.3%)	5 (31.2%)	0.39
Previous biologic treatment (switched)				
BLM	11 (35.5%)	3 (20.0%)	8 (50.0%)	0.14

Data are presented as means ± standard deviations or medians (interquartile ranges) unless otherwise indicated. SLEDAI, SLE Disease Activity Index 2000; BILAG, British Isles Lupus Assessment Group 2004 index; PSL, prednisolone; HCQ, hydroxychloroquine; IS, immunosuppressants; MMF, mycophenolate mofetil; MZR, mizoribine; AZA, azathioprine; MTX, methotrexate; TAC, tacrolimus; CsA, cyclosporine A; BLM, belimumab.

### 3.2 Clinical response

**Figure 2:**
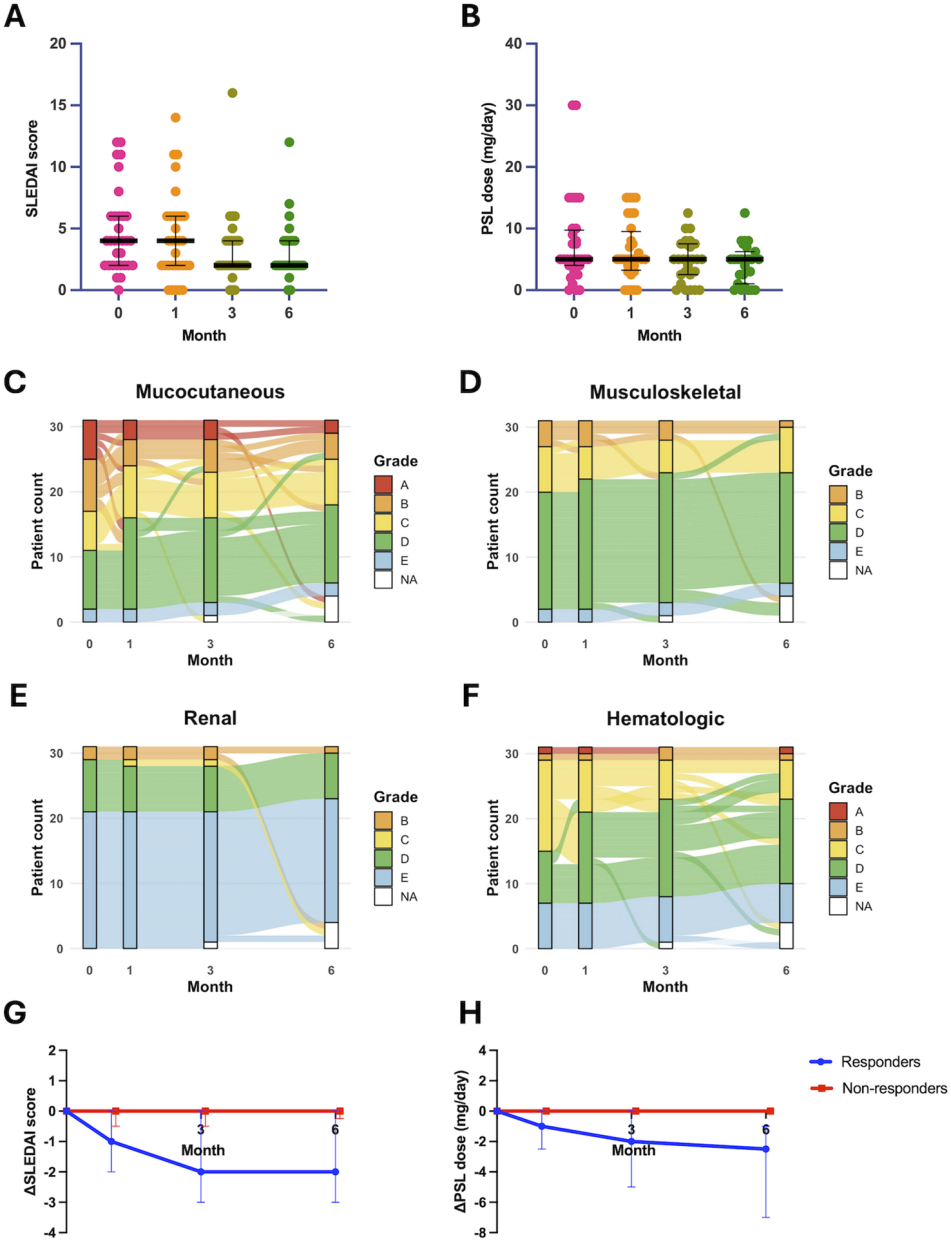
Clinical outcomes over 6 months. Changes in the SLEDAI score **(A)** and PSL dose (mg/day) **(B)** over 6 months. Dots represent individual patients; horizontal bars and whiskers indicate the median and interquartile range (IQR). BILAG domain grade transitions over 6 months in the mucocutaneous **(C)**, musculoskeletal **(D)**, renal **(E)**, and hematologic **(F)** domains. Sankey diagrams depict patient migration between BILAG grades (A-E/NA); bandwidth is proportional to the number of patients moving between grades. Change from baseline (Δ) in the SLEDAI score **(G)** and PSL dose (mg/day) **(H)** over 6 months by response group. Solid lines show group medians for responders **(blue)** and non-responders (red); vertical error bars indicate the IQR. SLEDAI, Systemic Lupus Erythematosus Disease Activity Index; PSL, prednisolone; BILAG, British Isles Lupus Assessment Group.

During the 6-month follow-up, the SLEDAI score declined progressively, whereas the median PSL dose remained largely unchanged (**Figures 2A, B**); individual trajectories are shown in **Supplementary Figure 2**. Organ-specific BILAG grades improved mainly in the mucocutaneous and hematologic domains (**Figures 2C–F**). Stratified analysis revealed that mucocutaneous improvement was predominantly driven by responders (**Supplementary Figures 3A–H**). Fifteen patients (48%) met the responder definition - achieving a BICLA response at month 6 or tapering GCs without flare - whereas 16 (52%) were categorized as non-responders (**Table 1**). As shown in **Figures 2G, H**, responders exhibited clear reductions in both SLEDAI score and PSL dose, whereas median values in non-responders showed minimal change in these parameters. Baseline disease duration, serological activity (anti-dsDNA titer, complement levels), autoantibody profile, organ involvement, and concomitant medications showed no statistically significant differences between the two groups (**Table 1**), indicating that they had comparable baseline clinical characteristics.

### 3.3 Changes in immune-cell subsets

**Figure 3:**
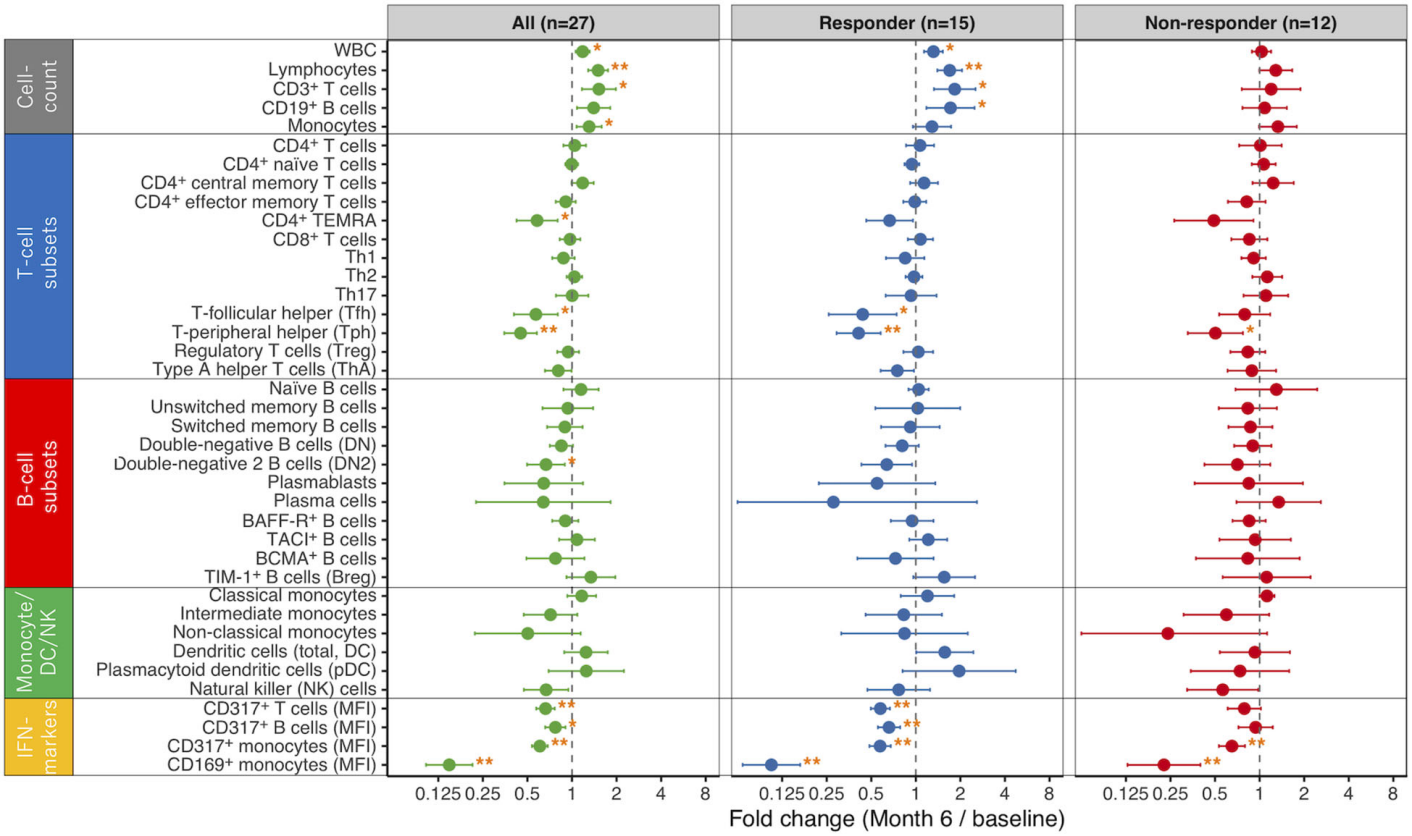
Immune biomarker changes over 6 months. Fold changes (month 6/baseline) in immune cell subset levels - including cell counts, proportions, and marker expression - are shown for the total cohort, as well as stratified by response (responders and non-responders). Points and error bars are back-transformed estimates with 95% CIs from analyses on the log_2_ scale; the x-axis is plotted on a log scale, and the dashed line at 1 indicates no change. Asterisks denote significance after Benjamini-Hochberg FDR adjustment (adjusted *P*): * 0.01 ≤ adjusted *P* < 0.05; ** adjusted *P* < 0.01. WBC, white blood cells; TEMRA, terminally differentiated effector memory CD4^+^ T cells re-expressing CD45RA; MFI, median fluorescence intensity.

Fold change from baseline to month 6 was calculated for each immune subset and compared across all patients, responders, and non-responders (**Figure 3**; numeric estimates and statistical results are summarized in **Supplementary Table 3**). Four patients lacking month 6 FACS data were excluded, leaving 27 patients (15 responders, 12 non-responders) for biomarker analysis (**Figure 1**). In the responder group, 10 immune-cell parameters showed significant modulation. Four absolute cell−count parameters—total white−blood−cell count, lymphocyte count, and CD3^+^ T−cell count, and CD19^+^ B-cell count—increased. Within the T−cell compartments, the frequencies of follicular helper T (Tfh) and peripheral helper T (Tph) cells decreased. No subset within the B-cell or monocyte/dendritic cell/natural killer (monocyte/DC/NK) lineages reached statistical significance. DN2 B cells showed a trend toward decrease, but the change did not reach statistical significance. For the type I IFN–responsive markers, significant decreases were observed in CD317 MFI on T cells and on B cells, and in both CD317 and CD169 MFI on monocytes - four IFN−related markers in total. In contrast, the non-responder group showed significant changes only in CD317 and CD169 MFI on monocytes, both type I IFN-responsive markers. No other immune-cell parameters exhibited statistically significant fold changes over the six−month period. Detailed time courses for these markers are provided in **Supplementary Figure 4**: CD169 MFI on monocytes decreased sharply by month 1, while the other markers showed gradual changes over the 6-month observation period.

### 3.4 Candidate biomarkers for response

**Figure 4:**
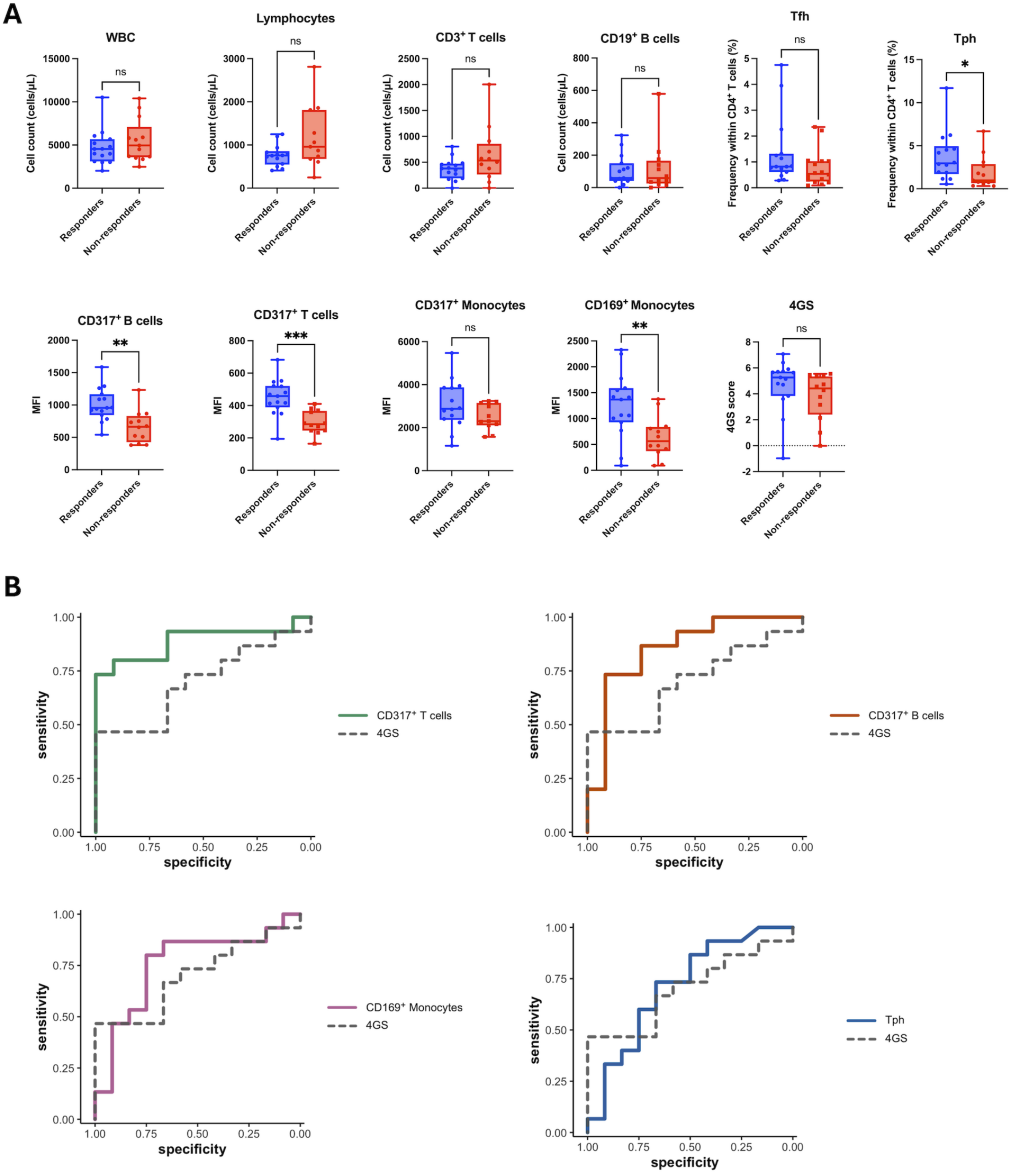
Baseline distributions and ROC performance of candidate biomarkers. **(A)** Baseline distributions of candidate biomarkers in responders and non-responders. Box plots show medians with interquartile ranges, whiskers indicate the range, and individual data points are overlaid. Asterisks denote significance: *P < 0.05; **P < 0.01; ***P < 0.001; ns, not significant. **(B)** ROC curves of the final candidate biomarkers predicting clinical response at month 6. The 4GS curve is shown as a dashed reference. AUCs with 95% CIs, optimal cut-offs, and DeLong tests are reported in **Table 2**.

Among the 10 IFN-related parameters that changed within responders (**Figure 3**), we compared baseline values between 15 responders and 12 non-responders included in the biomarker analysis (**Figure 4A**). We also evaluated the 4GS - an IFN-related gene signature previously reported to correlate with response to ANI (9). CD317 MFI on T-cells, CD317 MFI on B-cells, CD169 MFI on monocyte, and the frequency of Tph cells were significantly higher at baseline in responders. Notably, the 4GS showed a numerical trend toward higher baseline values in responders, but the difference did not reach statistical significance.

Biomarkers that differed at baseline (*P* < 0.05) - CD317 MFI on T-cells, CD317 MFI on B-cells, CD169 MFI on monocytes, and the frequency of Tph cells - were designated as final candidate biomarkers and evaluated by ROC analysis together with the 4GS (**Figure 4B**). CD317 MFI on T cells discriminated responders from non-responders and outperformed the 4GS (DeLong *P* = 0.044). CD317 MFI on B cells showed similarly good discrimination but did not differ significantly from the 4GS. CD169 MFI on monocytes and the frequency of Tph cells performed comparably to the 4GS. Sensitivity and specificity were calculated at the optimal cutoffs determined by Youden’s *J* index, and the corresponding cutoff units are presented in **Table 2**. Bootstrap-based internal validation with out-of-bag evaluation yielded an optimism-corrected AUC of 0.84 (bootstrap percentile interval 0.56–1.00).

**Table 2:** ROC analysis of final candidate biomarkers.

Biomarker	AUC (95% CI)	P-value	Cutoff	Sensitivity (%)	Specificity (%)
CD317^+^ T cells	0.889 (0.753–1.000)	0.044	411.5 (MFI)	73.3	100
CD317^+^ B cells	0.856 (0.701–1.000)	0.131	888 (MFI)	73.3	91.7
CD169^+^ Monocytes	0.756 (0.557–0.954)	0.487	889 (MFI)	80	75
Tph	0.714 (0.507–0.920)	0.893	1.835 (%)	73.3	66.7
4GS	0.700 (0.496–0.904)	—	5.592 (score)	46.7	100

Data are presented as areas under the receiver operating characteristic (ROC) curve (AUCs) with 95% confidence intervals (CIs). *P* values were obtained from paired DeLong tests versus the 4GS reference. Sensitivity and specificity were calculated at the optimal cutoffs determined by Youden’s J index; units for cutoffs are shown in parentheses. Tph, peripheral helper T cells; 4GS, 4-gene type I IFN signature; MFI, median fluorescence intensity.

Sensitivity analysis using the modified responder definition yielded consistent results (**Supplementary Table 4**); baseline CD317 MFI on T cells achieved an AUC of 0.786 (95% CI 0.590–0.981), which was higher than that of the 4GS (AUC 0.593; 95% CI 0.365–0.822). In additional sensitivity analyses, the 6-gene IFN signature (6GS) and each of the six individual ISG transcripts did not outperform baseline CD317 MFI on T cells (**Supplementary Table 5**; **Supplementary Figure 5**).

### 3.5 Relationship between final candidate biomarkers and clinical indices

**Figure 5:**
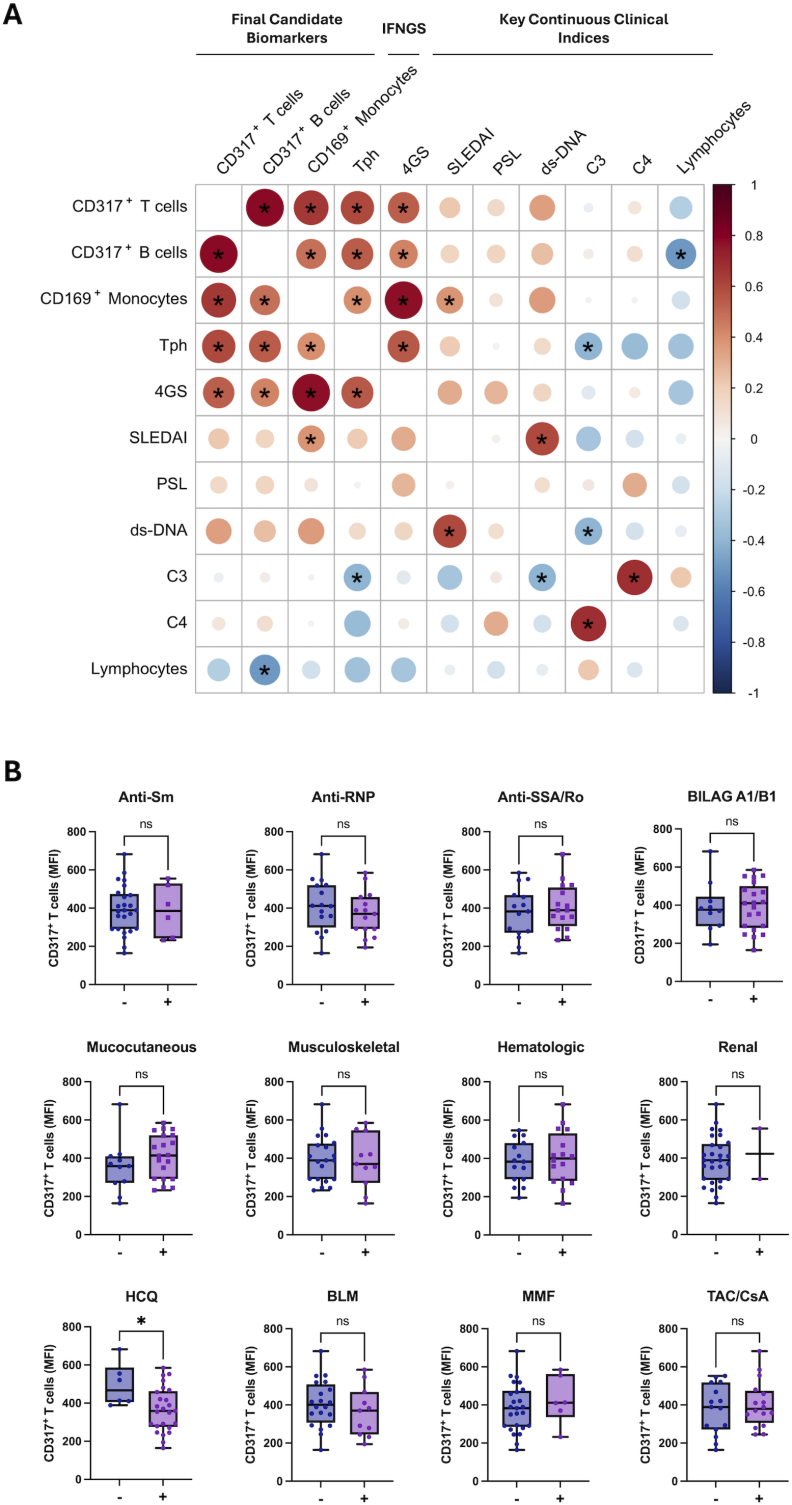
Clinical correlates of final candidate biomarkers. **(A)** Correlation matrix between final candidate biomarkers, the type I IFN gene signature (IFNGS; 4GS), and key continuous clinical indices. Circles encode Spearman correlations (color indicates sign and magnitude; size scales with |r|). Asterisks indicate two-sided P <0.05. **(B)** Distribution of CD317^+^ T-cell MFI across clinical categories. Box-and-whisker plots show medians with interquartile ranges and individual values. Asterisks denote significance (*P < 0.05; ns, not significant). Tph, T peripheral helper cells; 4GS, four-gene interferon score; SLEDAI, Systemic Lupus Erythematosus Disease Activity Index; PSL, prednisolone (daily dose); ds-DNA, anti-double-stranded DNA antibody; C3/C4, complement components; HCQ, hydroxychloroquine; BLM, belimumab; MMF, mycophenolate mofetil; TAC/CsA, tacrolimus/cyclosporine A; BILAG, British Isles Lupus Assessment Group.

For the four final candidate markers - CD317 MFI on T-cells, CD317 MFI on B-cells, CD169 MFI on monocytes, and the frequency of Tph cells - we assessed correlations with key continuous clinical indices (SLEDAI score, anti-dsDNA antibody titer, daily PSL dose, complement C3/C4 levels) as well as with the 4GS (**Figure 5A**). The four final markers were significantly and positively correlated with one another, and each showed a positive correlation with the 4GS. Although their associations with SLEDAI did not reach statistical significance, all four displayed a consistent positive trend. Conversely, all four markers were negatively correlated with absolute lymphocyte count, with the correlation reaching statistical significance for CD317 MFI on B cells.

Given its highest ROC AUC, CD317 MFI on T-cells was further examined across categorical variables, including autoantibody status (anti-Sm, anti-RNP, anti-SSA/Ro); presence of BILAG grade A or B involvement (A1/B1); major organ involvement (mucocutaneous, musculoskeletal, hematologic, or renal); concomitant medications at ANI initiation (HCQ or other immunosuppressants); and prior BLM treatment. CD317 expression did not differ across these categories except for patients receiving HCQ, who showed significantly lower CD317 MFI on T cells (**Figure 5B**).

## 4 Discussion

We evaluated the clinical response to ANI in relation to immune-cell subsets and type I interferon (IFN)–associated markers. Baseline CD317 expression on T cells predicted subsequent response as accurately as, or even more accurately than, the canonical four-gene IFN signature (4GS).

*Post hoc* analyses of pivotal ANI trials have reported superior outcomes in patients with elevated whole-blood IFNGS (10). Consistent with these findings, the PBMC-derived 4GS predicted treatment response well in our cohort. However, IFNGS values depend strongly on the cellular composition of the analyzed sample: in whole blood, neutrophils account for 40-70% of leukocytes (29), and the measured signal therefore primarily reflects interferon-stimulated gene expression in neutrophils. PBMCs, by contrast, are enriched for lymphocytes and monocytes. Large-scale single-cell transcriptomic analyses encompassing 27 immune-cell subsets have demonstrated that upregulation of IFN-α response genes is most pronounced in T- and B-cell clusters and correlates closely with high-activity in SLE (30). These observations suggest that PBMC- or lymphocyte-based 4GS measurements may more accurately capture the type I interferon signal that is directly linked to disease activity.

Despite its utility, routine measurement of the 4GS requires gene-expression assays and is not entirely specific for type I IFN, as type II and type III IFNs can also induce these transcripts. Indeed, reporter-cell bioassays detect high type I IFN bioactivity in only about one-third of patients with elevated 4GS values (11). While MX1 and IFIT1 have been used as readouts in a functional assay designed to capture type I IFN activity (31, 32), our sensitivity analysis evaluating six representative ISGs individually showed that none achieved higher discriminative performance than baseline CD317 MFI on T cells (**Supplementary Table 5**; **Supplementary Figure 5**). Notably, MX1 showed a numerically higher AUC among the individual transcripts, but its performance remained lower than that of CD317 MFI on T cells. Direct protein quantification is equally challenging: multiple IFN-α subtypes and IFN-β must be measured; total circulating concentrations do not necessarily reflect functional activity (14, 33); even ultrasensitive digital ELISA (Simoa) tends to favor certain subtypes (34); and reporter-cell methods are time-consuming and labor-intensive (32). Thus, our findings - that flow-cytometric measurement of the single surface marker CD317 predicts response to ANI at least as well as the transcript-based IFNGS - support the potential of a simple and readily implementable assay for assessing disease activity and therapeutic response.

CD317 (BST-2), initially identified as an interferon-inducible restriction factor that inhibits retrovirus release (35), is also a cell surface marker exquisitely sensitive to type I IFNs. CD317 expression was remarkably induced in human CD4^+^ T, CD8^+^ T, and monocytes by type I IFN but not type II IFN (36). Notably, despite higher basal CD317 expression on monocytes, lymphocyte subsets show a greater dynamic range of CD317 induction in response to type I IFN stimulation, suggesting that lymphocyte CD317 may better capture inter-individual variability in type I IFN responsiveness (15). In SLE, IFN signaling in T and B lymphocytes has been suggested to associate more closely with disease activity than signaling in myeloid cells such as monocytes (30). CD317 therefore merits attention as a readout of lymphocyte-driven type I IFN responses. Consistent with this notion, baseline CD317 expression on lymphocytes in the present analysis predicted the response to ANI more accurately than its expression on monocytes. These findings suggest that treatment response is likely reflected more precisely by IFN activity detected in lymphocytes than by that in monocytes. A plausible explanation is that CD317 on lymphocytes provides a downstream, protein-level readout of the net biological impact of type I IFN—integrating both upstream IFN exposure and inter-individual differences in cellular responsiveness—thereby providing a more integrative signal for treatment response assessment than monocyte-based readouts. Supporting this lymphocyte focus, CD317 on memory B cells has been reported to correlate with the number of active BILAG domains and to predict impending flares (15). Nonetheless, the basis for the differential performance of CD317 between T and B cells remains unclear and warrants further investigation in larger cohorts and mechanistic studies. Reanalysis of a publicly available SLE scRNA-seq dataset (37) further showed that BST2/CD317 expression at the transcript level on both lymphocytes and monocytes correlated strongly with the IFNGS, but only lymphocyte CD317 expression - particularly on T cells - showed a trend toward association with the SLEDAI (**Supplementary Figure 6**). Taken together, the strong predictive performance of baseline CD317 expression on T and B cells, coupled with the limited utility of monocyte-derived markers observed in our study, aligns well with these prior observations.

In the correlation analysis, baseline CD317 expression on lymphocytes reflected type I IFN-driven activity but was only partially captured by established clinical indices. CD317 MFI on T and B cells correlated positively with each other and with the four-gene IFN signature (4GS). By contrast, their associations with the SLEDAI did not reach statistical significance, although the direction of the effect was positive. CD317 MFI on B cells was inversely correlated with absolute lymphocyte count, consistent with the recognized link between heightened type I IFN activity and lymphopenia. CD317 MFI on T cells showed no association with autoantibody profiles, and organ-specific activity defined by individual BILAG domains including BILAG A1/B1 in this study. These findings do not imply that CD317 MFI on T cells is an independent predictor beyond the 4GS or clinical activity indices such as SLEDAI. Rather, CD317 MFI on T cells may serve as a practical protein-level surrogate reflecting patient-specific type I IFN responsiveness that is not fully captured by conventional clinical indices. Notably, baseline SLEDAI did not differ between responders and non-responders. On the other hand, El-Sherbiny et al. reported that CD317 expression on memory B cells correlated positively with the composite BILAG score (15). Differences in the target population (memory B cells *vs* total T cells), fluorescence metric (mean *vs* median MFI), and our cohort’s milder disease activity likely explain this apparent discrepancy. Nevertheless, the superior predictive performance of CD317 in our ROC analysis supports its specificity for type I IFN activity, indicating that the two studies are substantively concordant.

Interestingly, in the present study, CD317^+^ T cells were decreased only in patients who received HCQ as premedication, but not in those treated with immunosuppressive agents such as mycophenolate mofetil, calcineurin inhibitors, or BLM. This finding underscores once again the fundamental importance of HCQ as a cornerstone therapy in SLE. Conversely, in patients with SLE who are unable to use HCQ for any reason, ANI may represent a valuable therapeutic option capable of modulating the IFN gene signature (IFNGS) during both the induction and maintenance phases of remission. This potential role of ANI warrants further investigation in a larger cohort of patients to confirm its clinical relevance.

Analysis of samples from patients who responded well to ANI suggested that type I IFN blockade affects a broad range of immune subsets; however, most molecules lacked sufficient baseline predictive ability. In addition to CD317, two immunologic parameters - the surface marker CD169 (SIGLEC-1) and the frequency of Tph subset - retained some association with response to ANI. Notably, CD169 MFI on monocytes showed the largest on-treatment fold change in responders, and both markers correlated positively with the 4GS, indicating their ability to sensitively identify patients with heightened type I IFN activity. Monocytes express much higher levels of IFNAR compared to T cells and B cells, and therefore exhibit stronger STAT1 phosphorylation and induction of ISGs in response to type I IFN stimulation (38). Like CD317, CD169 is an IFN-inducible molecule, but its expression is largely restricted to the monocyte/macrophage lineage (39), and may therefore underrepresent lymphocyte-centered processes. Nevertheless, its dynamic behavior is informative, as a decrease in CD169 expression on monocyte after ANI treatment has been shown to parallel clinical improvement in cutaneous lupus (40). Conversely, Tph cells - whose developmental pathway is distinct from that of classical Tfh cells - have been reported to be promoted by type I IFN signaling during differentiation (41, 42); accordingly, the decline in Tph frequency observed after ANI initiation is biologically coherent. Although CD169 and Tph underperformed CD317 as individual predictors, larger cohorts and multivariable models are warranted to determine their respective contributions and to test whether combining multiple markers can enhance predictive accuracy.

Our findings indicate that assessing baseline CD317 expression on T cells before initiating ANI may enable clinicians to predict therapeutic response in advance. In SLE, baseline serum BAFF levels have already been shown to predict the efficacy of BLM (43–45). Establishing CD317 or related indices might eventually assist in more precise selection between ANI and other biologic agents. Given that type I IFN signaling is also implicated in other autoimmune disorders, this concept might extend to broader indications for ANI and to more personalized treatment strategies in those diseases. The generalizability of the results may be influenced by several limitations inherent in this study. First, the sample size was small, limiting statistical power; ideally, adjustment for additional variables in a larger, multicenter, multiracial study would yield more precise results. External validation in an independent cohort would be preferred, but an appropriate external dataset is not currently available. Bootstrap-based internal validation with out-of-bag evaluation was conducted to estimate an optimism-corrected AUC, which supported the relative robustness of the signal within the examined candidate set. Nevertheless, uncertainty remained substantial, as reflected by the wide bootstrap interval. Accordingly, these findings should be interpreted as exploratory and warrant confirmation through external validation and further internal validation in larger, independent cohorts. Second, baseline disease activity was relatively low in this cohort, which may limit the sensitivity of achievement-based composite response endpoints. Nonetheless, the main findings were generally consistent across response definitions. Third, follow-up was limited to 6 months, which precluded assessment of long-term outcomes such as sustained remission or organ-damage progression; external validation in longer prospective cohorts is therefore warranted. Fourth, because this was a single-arm prospective study without a standardized protocol for concomitant immunosuppressant or GC use, physician discretion may have introduced bias. Finally, this study did not address the functional significance of CD317^+^ T and B cells in the pathogenesis of SLE, which remains to be elucidated. Future investigations should employ randomized controlled trial designs initiated at ANI treatment onset and apply standardized dose-adjustment and steroid-tapering algorithms to enable robust causal inference. By overcoming these limitations - and by integrating comprehensive analyses of cell surface markers such as CD317 with IFNGS profiles - we may move closer to realizing precision medicine in SLE.

In conclusion, our study identifies higher baseline CD317 expression on T cells as a robust indicator of clinical responsiveness to ANI in SLE, outperforming IFNGS in predictive accuracy. Beyond its strong performance as a response predictor, CD317 offers practical advantages, including feasibility for T cell-focused assays and potential adaptability to clinical flow cytometry-based testing, highlighting its promise as a clinically actionable companion biomarker. To facilitate a holistic interpretation of the biomarker-driven prediction model, we provide a consolidated visual overview of the study design, core biomarker findings, and the immunological features that distinguish ANI responders from non-responders (**Supplementary Figure 7**). Future multi-cohort validation and translational implementation studies will be critical to further establish the robustness, generalizability, and real-world clinical utility of CD317 in guiding personalized ANI therapy for patients with SLE.

## Data Availability

The original contributions presented in the study are included in the article/**Supplementary Material**. Further inquiries can be directed to the corresponding authors.

## References

[1] Fanouriakis A Tziolos N Bertsias G Boumpas DT . Update on the diagnosis and management of systemic lupus erythematosus. Ann Rheum Dis. (2021) 80:14–25. doi: 10.1136/annrheumdis-2020-218272, PMID: 33051219

[2] Dörner T Furie R . Novel paradigms in systemic lupus erythematosus. Lancet. (2019) 393:2344–58. doi: 10.1016/S0140-6736(19)30546-X, PMID: 31180031

[3] Tsokos GC . Autoimmunity and organ damage in systemic lupus erythematosus. Nat Immunol. (2020) 21:605–14. doi: 10.1038/s41590-020-0677-6, PMID: 32367037 PMC8135909

[4] Fasano S Milone A Nicoletti GF Isenberg DA Ciccia F . Precision medicine in systemic lupus erythematosus. Nat Rev Rheumatol. (2023) 19:331–42. doi: 10.1038/s41584-023-00948-y, PMID: 37041269

[5] Bennett L Palucka AK Arce E Cantrell V Borvak J Banchereau J . Interferon and granulopoiesis signatures in systemic lupus erythematosus blood. J Exp Med. (2003) 197:711–23. doi: 10.1084/jem.20021553, PMID: 12642603 PMC2193846

[6] Baechler EC Batliwalla FM Karypis G Gaffney PM Ortmann WA Espe KJ . Interferon-inducible gene expression signature in peripheral blood cells of patients with severe lupus. Proc Natl Acad Sci U.S.A. (2003) 100:2610–5. doi: 10.1073/pnas.0337679100, PMID: 12604793 PMC151388

[7] Arazi A Rao DA Berthier CC Davidson A Liu Y Hoover PJ . The immune cell landscape in kidneys of patients with lupus nephritis. Nat Immunol. (2019) 20:902–14. doi: 10.1038/s41590-019-0398-x, PMID: 31209404 PMC6726437

[8] Furie RA Morand EF Bruce IN Manzi S Kalunian KC Vital EM . Type I interferon inhibitor anifrolumab in active systemic lupus erythematosus (TULIP-1): a randomised, controlled, phase 3 trial. Lancet Rheumatol. (2019) 1:e208–19. doi: 10.1016/S2665-9913(19)30076-1 38229377

[9] Morand EF Furie R Tanaka Y Bruce IN Askanase AD Richez C . Trial of anifrolumab in active systemic lupus erythematosus. N Engl J Med. (2020) 382:211–21. doi: 10.1056/NEJMoa1912196 31851795

[10] Vital EM Merrill JT Morand EF Furie RA Bruce IN Tanaka Y . Anifrolumab efficacy and safety by type I interferon gene signature and clinical subgroups in patients with SLE: *post hoc* analysis of pooled data from two phase III trials. Ann Rheum Dis. (2022) 81:951–61. doi: 10.1136/annrheumdis-2021-221425, PMID: 35338035 PMC9213795

[11] Gómez-Bañuelos E Goldman DW Andrade V Darrah E Petri M Andrade F . Uncoupling interferons and the interferon signature explains clinical and transcriptional subsets in SLE. Cell Rep Med. (2024) 5:101569. doi: 10.1016/j.xcrm.2024.101569, PMID: 38744279 PMC11148857

[12] Zhou Z Hamming OJ Ank N Paludan SR Nielsen AL Hartmann R . Type III Interferon (IFN) Induces a Type I IFN-Like Response in a Restricted Subset of Cells through Signaling Pathways Involving both the Jak-STAT Pathway and the Mitogen-Activated Protein Kinases. J Virol. (2007) 81:7749–58. doi: 10.1128/JVI.02438-06, PMID: 17507495 PMC1933366

[13] Sekrecka A Kluzek K Sekrecki M Boroujeni ME Hassani S Yamauchi S . Time-dependent recruitment of GAF, ISGF3 and IRF1 complexes shapes IFNα and IFNγ-activated transcriptional responses and explains mechanistic and functional overlap. Cell Mol Life Sci. (2023) 80:187. doi: 10.1007/s00018-023-04830-8, PMID: 37347298 PMC10287828

[14] Rodero MP Decalf J Bondet V Hunt D Rice GI Werneke S . Detection of interferon alpha protein reveals differential levels and cellular sources in disease. J Exp Med. (2017) 214:1547–55. doi: 10.1084/jem.20161451, PMID: 28420733 PMC5413335

[15] El-Sherbiny YM Md Yusof MY Psarras A Hensor EMA Kabba KZ Dutton K . B cell tetherin: A flow cytometric cell-specific assay for response to type I interferon predicts clinical features and flares in systemic lupus erythematosus. Arthritis Rheumatol. (2020) 72:769–79. doi: 10.1002/art.41187, PMID: 31804007 PMC8653884

[16] Biesen R Demir C Barkhudarova F Grün JR Steinbrich-Zöllner M Backhaus M . Sialic acid-binding Ig-like lectin 1 expression in inflammatory and resident monocytes is a potential biomarker for monitoring disease activity and success of therapy in systemic lupus erythematosus. Arthritis Rheumatol. (2008) 58:1136–45. doi: 10.1002/art.23404, PMID: 18383365

[17] Rönnblom L Elkon KB . Cytokines as therapeutic targets in SLE. Nat Rev Rheumatol. (2010) 6:339–47. doi: 10.1038/nrrheum.2010.64, PMID: 20440285

[18] Aringer M Costenbader K Daikh D Brinks R Mosca M Ramsey-Goldman R . European league against rheumatism/American college of rheumatology classification criteria for systemic lupus erythematosus. Arthritis Rheumatol. (2019) 71:1400–12. doi: 10.1002/art.40930, PMID: 31385462 PMC6827566

[19] Gladman DD Ibañez D Urowitz MB . Systemic lupus erythematosus disease activity index 2000. J Rheumatol. (2002) 29:288–91. 11838846

[20] Isenberg DA Rahman A Allen E Farewell V Akil M Bruce IN . Development and initial validation of an updated version of the British Isles Lupus Assessment Group’s disease activity index for patients with systemic lupus erythematosus. Rheumatol (Oxford). (2005) 44:902–6. doi: 10.1093/rheumatology/keh624, PMID: 15814577

[21] Wallace DJ Kalunian K Petri MA Strand V Houssiau FA Pike M . Efficacy and safety of epratuzumab in patients with moderate/severe active systemic lupus erythematosus: results from EMBLEM, a phase IIb, randomised, double-blind, placebo-controlled, multicentre study. Ann Rheum Dis. (2014) 73:183–90. doi: 10.1136/annrheumdis-2012-202760, PMID: 23313811 PMC3888603

[22] Isenberg DA Allen E Farewell V D’Cruz D Alarcón GS Aranow C . An assessment of disease flare in patients with systemic lupus erythematosus: a comparison of BILAG 2004 and the flare version of SELENA. Ann Rheum Dis. (2011) 70:54–9. doi: 10.1136/ard.2010.132068, PMID: 20833737

[23] Maecker HT McCoy JP Nussenblatt R . Standardizing immunophenotyping for the human immunology project. Nat Rev Immunol. (2012) 12:191–200. doi: 10.1038/nri3158, PMID: 22343568 PMC3409649

[24] Jenks SA Cashman KS Zumaquero E Marigorta UM Patel AV Wang X . Distinct effector B cells induced by unregulated Toll-like receptor 7 contribute to pathogenic responses in systemic lupus erythematosus. Immunity. (2018) 49:725–39.e6. doi: 10.1016/j.immuni.2018.08.015, PMID: 30314758 PMC6217820

[25] Cherukuri A Mohib K Rothstein DM . Regulatory B cells: TIM-1, transplant tolerance, and rejection. Immunol Rev. (2021) 299:31–44. doi: 10.1111/imr.12933, PMID: 33484008 PMC7968891

[26] Goto M Takahashi H Yoshida R Itamiya T Nakano M Nagafuchi Y . Age-associated CD4+ T cells with B cell–promoting functions are regulated by ZEB2 in autoimmunity. Sci Immunol. (2024) 9:eadk1643. doi: 10.1126/sciimmunol.adk1643, PMID: 38330141

[27] Rao DA Gurish MF Marshall JL Slowikowski K Fonseka CY Liu Y . Pathologically expanded peripheral T helper cell subset drives B cells in rheumatoid arthritis. Nature. (2017) 542:110–4. doi: 10.1038/nature20810, PMID: 28150777 PMC5349321

[28] Burska A Rodríguez-Carrio J Biesen R Dik WA Eloranta ML Cavalli G . Type I interferon pathway assays in studies of rheumatic and musculoskeletal diseases: a systematic literature review informing EULAR points to consider. RMD Open. (2023) 9:e002876. doi: 10.1136/rmdopen-2022-002876, PMID: 36863752 PMC9990675

[29] Hsu AY Peng Z Luo H Loison F . Isolation of human neutrophils from whole blood and buffy coats. J Vis Exp. (2021) 175:e62837. doi: 10.3791/62837, PMID: 34605812

[30] Nakano M Ota M Takeshima Y Iwasaki Y Hatano H Nagafuchi Y . Distinct transcriptome architectures underlying lupus establishment and exacerbation. Cell. (2022) 185:3375–89.e21. doi: 10.1016/j.cell.2022.07.021, PMID: 35998627

[31] Oke V Gunnarsson I Dorschner J Eketjäll S Zickert A Niewold TB . High levels of circulating interferons type I, type II and type III associate with distinct clinical features of active systemic lupus erythematosus. Arthritis Res Ther. (2019) 29:21. doi: 10.1186/s13075-019-1878-y, PMID: 31036046 PMC6489203

[32] Hua J Kirou K Lee C Crow MK . Functional assay of type I interferon in systemic lupus erythematosus plasma and association with anti-RNA binding protein autoantibodies. Arthritis Rheumatol. (2006) 54:1906–16. doi: 10.1002/art.21890, PMID: 16736505

[33] Moll HP Maier T Zommer A Lavoie T Brostjan C . The differential activity of interferon-α subtypes is consistent among distinct target genes and cell types. Cytokine. (2011) 53:52–9. doi: 10.1016/j.cyto.2010.09.006, PMID: 20943413 PMC3020287

[34] Mathian A Mouries-Martin S Dorgham K Devilliers H Barnabei L Ben Salah E . Monitoring disease activity in systemic lupus erythematosus with single-molecule array digital enzyme-linked immunosorbent assay quantification of serum interferon-α. Arthritis Rheumatol. (2019) 71:756–65. doi: 10.1002/art.40792, PMID: 30507062

[35] Neil SJD Zang T Bieniasz PD . Tetherin inhibits retrovirus release and is antagonized by HIV-1 Vpu. Nature. (2008) 451:425–30. doi: 10.1038/nature06553, PMID: 18200009

[36] Bego MG Mercier J Cohen ÉA . Virus-activated interferon regulatory factor 7 upregulates expression of the interferon-regulated BST2 gene independently of interferon signaling. J Virol. (2012) 86:3513–27. doi: 10.1128/JVI.06971-11, PMID: 22301143 PMC3302510

[37] Nehar-Belaid D Hong S Marches R Chen G Bolisetty M Baisch J . Mapping systemic lupus erythematosus heterogeneity at the single-cell level. Nat Immunol. (2020) 21:1094–106. doi: 10.1038/s41590-020-0743-0, PMID: 32747814 PMC7442743

[38] van Boxel-Dezaire AHH Zula JA Xu Y Ransohoff RM Jacobberger JW Stark GR . Major differences in the responses of primary human leukocyte subsets to IFN-β. J Immunol. (2010) 185:5888–99. doi: 10.4049/jimmunol.0902314, PMID: 20956346 PMC3244975

[39] Crocker PR Paulson JC Varki A . Siglecs and their roles in the immune system. Nat Rev Immunol. (2007) 7:255–66. doi: 10.1038/nri2056, PMID: 17380156

[40] Carter LM Wigston Z Laws P Vital EM . Rapid efficacy of anifrolumab across multiple subtypes of recalcitrant cutaneous lupus erythematosus parallels changes in discrete subsets of blood transcriptomic and cellular biomarkers. Br J Dermatol. (2023) 189:210–8. doi: 10.1093/bjd/ljad089, PMID: 36944572

[41] Law C Wacleche VS Cao Y Pillai A Sowerby J Hancock B . Interferon subverts an AHR-JUN axis to promote CXCL13+ T cells in lupus. Nature. (2024) 631:857–66. doi: 10.1038/s41586-024-07627-2, PMID: 38987586 PMC11628166

[42] Bocharnikov AV Keegan J Wacleche VS Cao Y Fonseka CY Wang G . PD-1hiCXCR5- T peripheral helper cells promote B cell responses in lupus via MAF and IL-21. JCI Insight. (2019) 4:e130062. doi: 10.1172/jci.insight.130062, PMID: 31536480 PMC6824311

[43] Piantoni S Regola F Masneri S Merletti M Lowin T Airò P . Characterization of B- and T-cell compartment and B-cell-related factors belonging to the TNF/TNFR superfamily in patients with clinically active systemic lupus erythematosus: baseline BAFF serum levels are the strongest predictor of response to belimumab after twelve months of therapy. Front Pharmacol. (2021) 12:666971. doi: 10.3389/fphar.2021.666971, PMID: 34093196 PMC8176088

[44] Roth DA Thompson A Tang Y Hammer AE Molta CT Gordon D . Elevated BLyS levels in patients with systemic lupus erythematosus: associated factors and responses to belimumab. Lupus. (2016) 25:346–54. doi: 10.1177/0961203315604909, PMID: 26385220 PMC4785993

[45] Wilkinson C Henderson RB Jones-Leone AR Flint SM Lennon M Levy RA . The role of baseline BLyS levels and type I interferon-inducible gene signature status in determining belimumab response in systemic lupus erythematosus: a *post hoc* meta-analysis. Arthritis Res Ther. (2020) 22:102. doi: 10.1186/s13075-020-02177-0, PMID: 32366280 PMC7197114

