# Supplementary material for: High baseline CD317 expression on T cells predicts favorable anifrolumab response in systemic lupus erythematosus
Source: Front Immunol. 2026 Mar 26;17:1756139. doi: 10.3389/fimmu.2026.1756139 (PMC13062285; doi:10.3389/fimmu.2026.1756139)
Supplement: Supplementary file 1 [file DataSheet1.pdf]

Supplementary Material

1 Supplementary Figures

1.1 Supplementary Figure S1

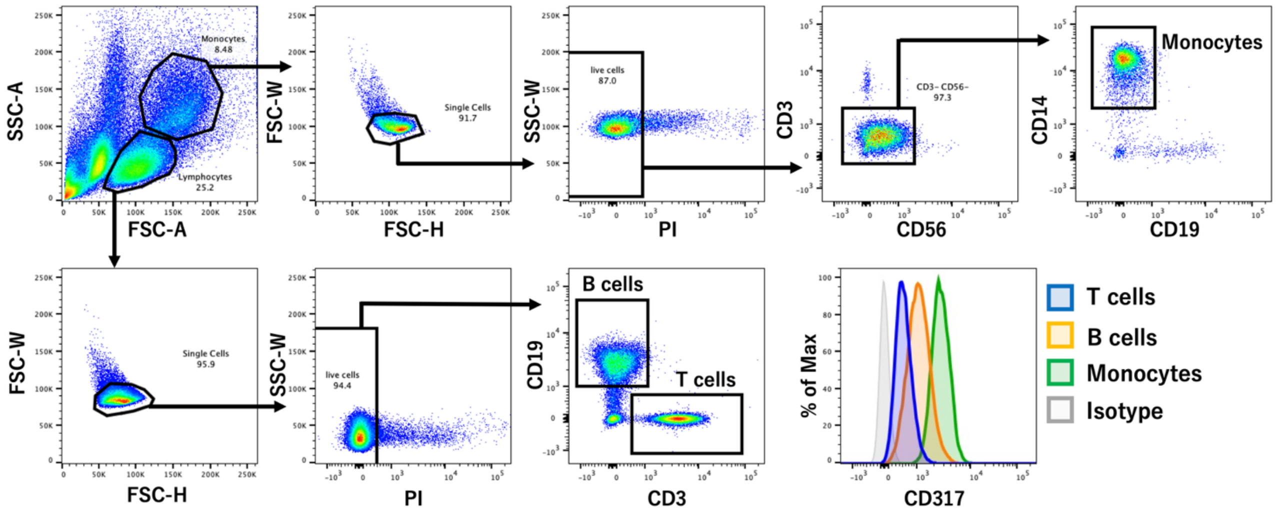

Representative gating strategy and CD317 histograms. Representative plots show the gating strategy for major subsets and overlaid CD317 histograms with the corresponding isotype control. PBMCs were gated by FSC/SSC, followed by singlet gating (FSC-based gate shown; an additional SSC-based singlet gate was applied but not shown) and live-cell selection (PI–). T cells ($CD3^{+}$), B cells ($CD19^{+}$), and monocytes ($CD14^{+}$ within $CD3^{-}CD19^{-}CD56^{-}$ cells) are shown.

1.2 Supplementary Figure S2

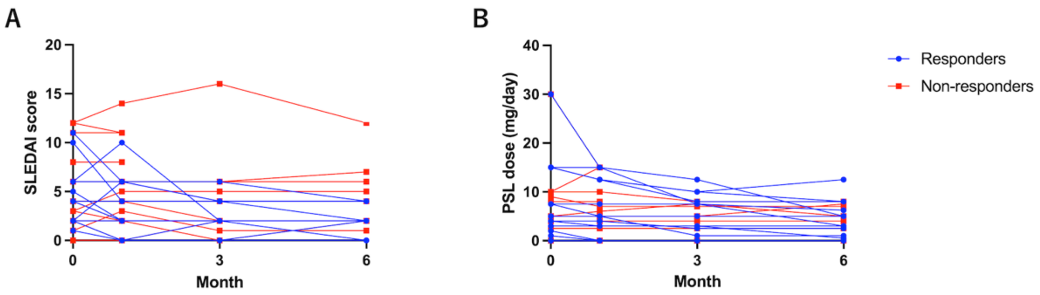

Changes in the SLEDAI score (A) and PSL dose (mg/day) (B) over 6 months. Spaghetti plots depict individual patient trajectories from baseline (month 0) to month 6. Responders are shown in blue and non-responders in red.

1.3 Supplementary Figure S3

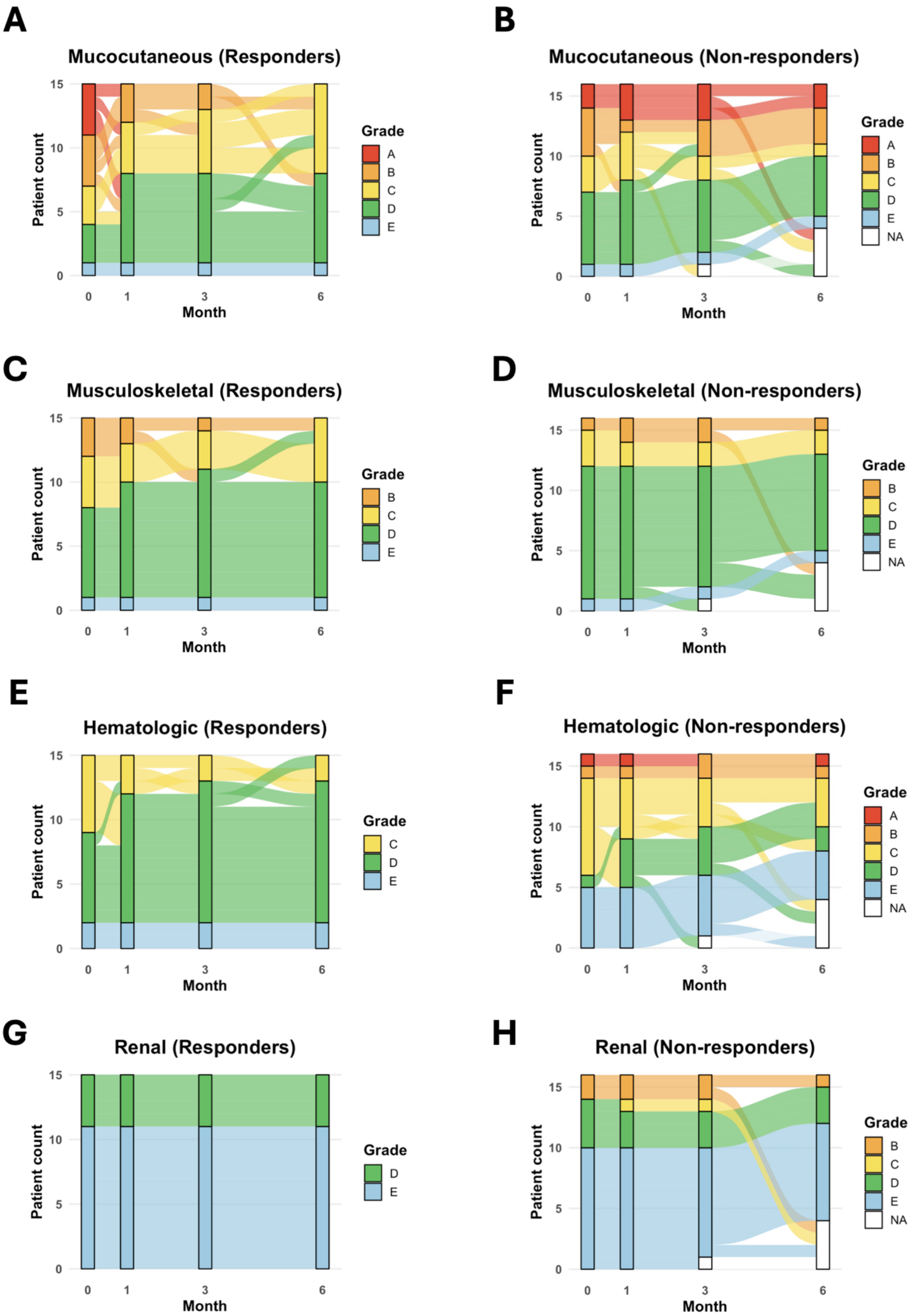

Stratified BILAG domain grade transitions over 6 months by response group. BILAG domain grade transitions over 6 months stratified by response status in the mucocutaneous (A, B), musculoskeletal (C, D), hematologic (E, F), and renal (G, H) domains. Panels show responders (A, C, E, G) and non-responders (B, D, F, H). Sankey diagrams depict patient migration between BILAG grades (A–E/NA) at months 0, 1, 3, and 6; bandwidth is proportional to the number of patients moving between grades. BILAG, British Isles Lupus Assessment Group.

1.4 Supplementary Figure S4

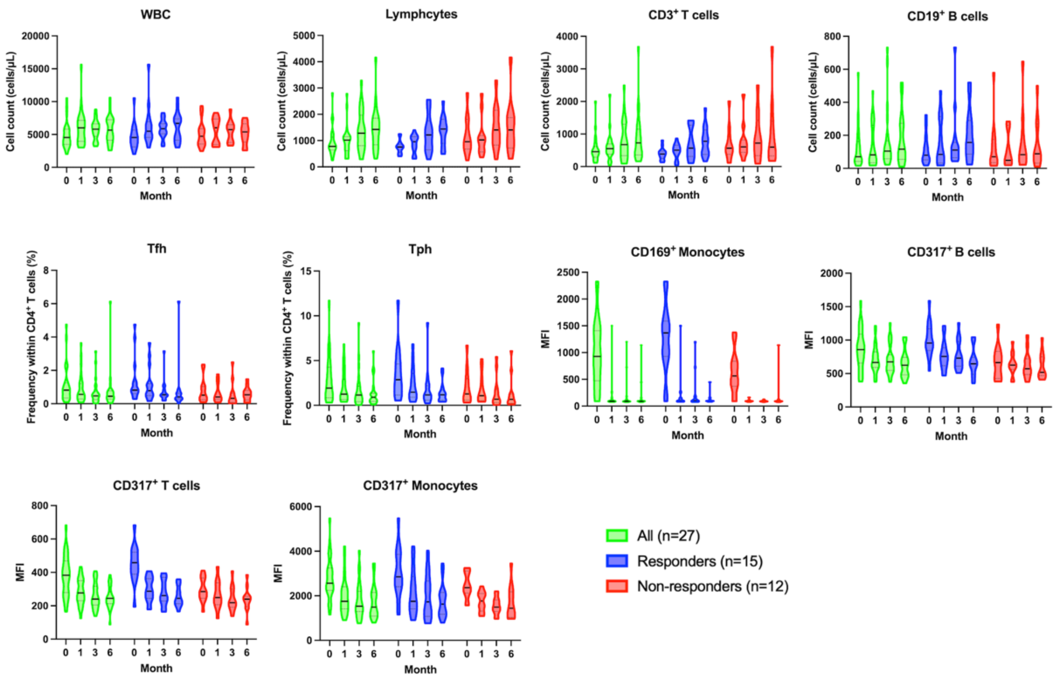

Temporal changes in the 10 candidate biomarkers over 6 months. Separated violin plots display the distribution of each biomarker at baseline (0) and months 1, 3, 6 for the entire cohort (All, green; n=27) and for patients stratified as responders (blue; n=15) or non-responders (red; n=12). The width of each violin reflects kernel-density estimates; the horizontal line within each violin denotes the median value.

1.5 Supplementary Figure S5

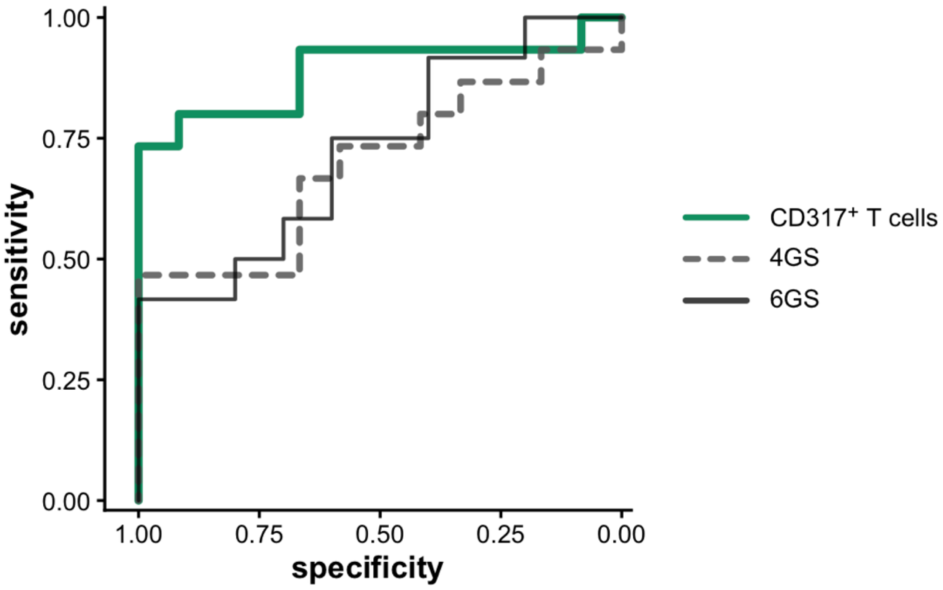

ROC curves for baseline CD317 expression on T cells and interferon gene signatures. The ROC curve for baseline CD317 MFI on T cells is shown together with the four-gene IFN signature (4GS; dashed gray line) and the six-gene IFN signature (6GS; solid black line) as sensitivity analyses. AUCs with 95% CIs are reported in Supplementary Table S5.

1.6 Supplementary Figure S6

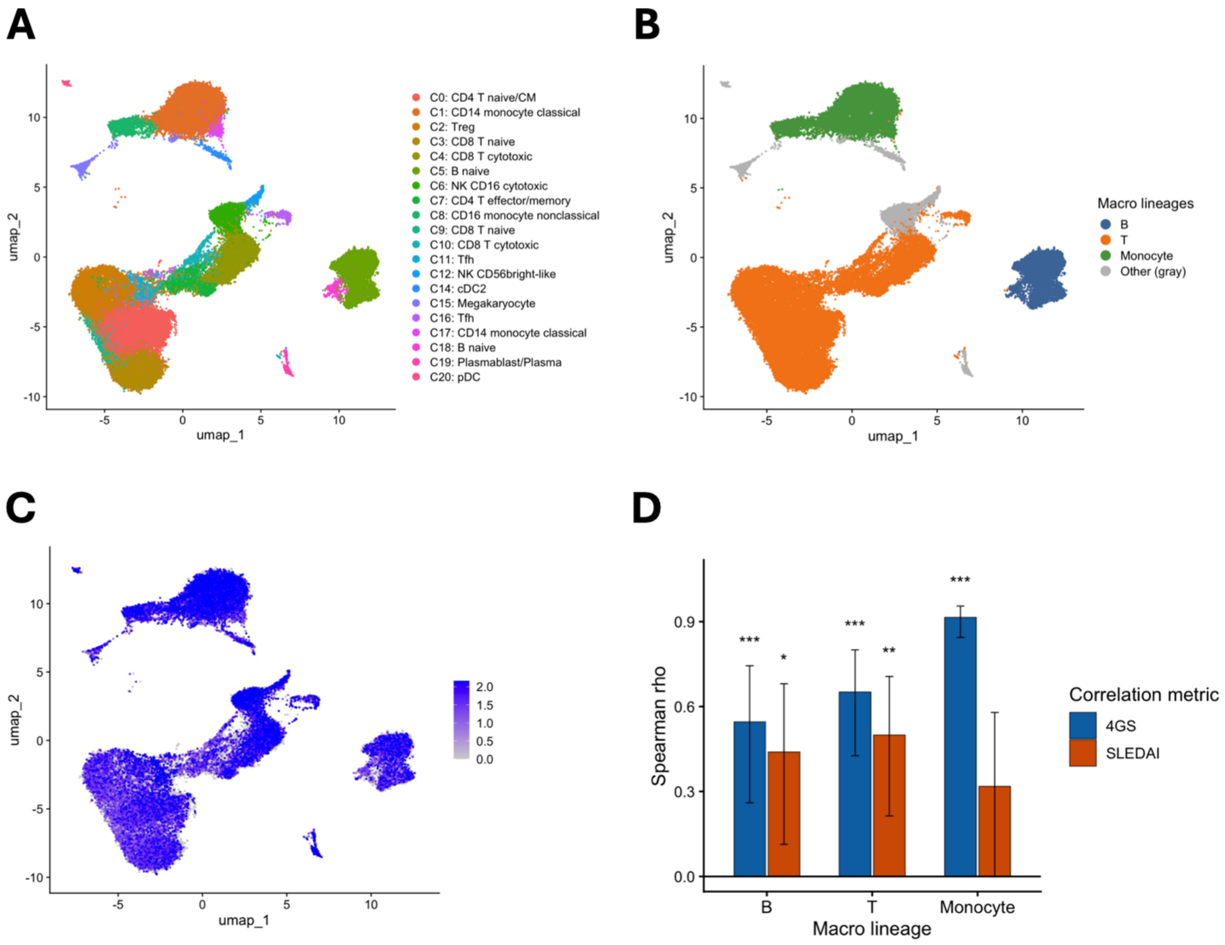

BST2/CD317 expression across PBMC lineages and its associations with the 4GS and SLEDAI in a public SLE scRNA-seq dataset. UMAP of PBMCs with canonical subset annotations is shown in (A) (erythroid cells excluded from display only), and the same UMAP colored by macro lineages (B, T, and monocytes; other lineages in gray) is shown in (B). BST2 expression projected onto the UMAP is shown in (C) (values clipped to the 5th–95th percentiles). Spearman correlations (rho ± 95% CI) between macro-lineage–level BST2 expression (per-sample median within each macro lineage; samples with ≥20 cells per lineage) and the 4GS (blue) or SLEDAI (orange) are shown in (D); *, **, *** indicate BH-adjusted $P<0.05$, 0.01, 0.001, respectively. PBMC, peripheral blood mononuclear cells; UMAP, Uniform Manifold Approximation and Projection; 4GS, 4-gene type I IFN signature.

1.7 Supplementary Figure S7

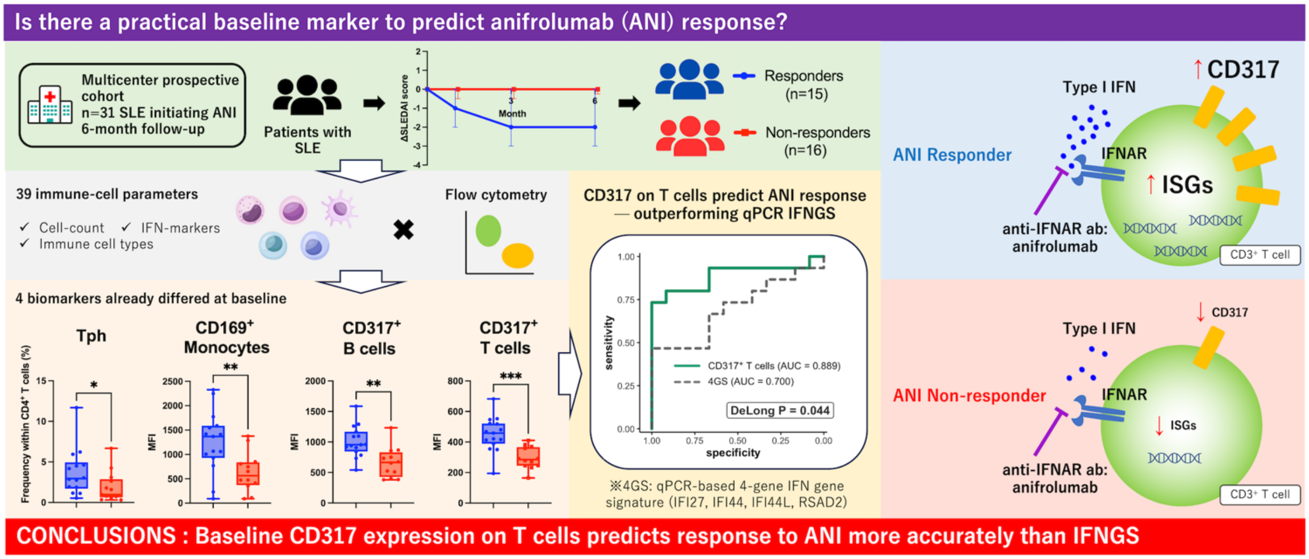

Graphical summary of the study design, key biomarker findings, and immunological features distinguishing anifrolumab responders from non-responders.

2 Supplementary Tables

2.1 Supplementary Table S1 (Excel): Antibodies and reagents used for flow-cytometry panels

Antibody panels used for immunophenotyping at baseline and follow-up. For each reagent, the fluorochrome, target marker, clone, and supplier are listed. All panels included a viability dye (propidium iodide, PI; BioLegend). PI was acquired in the PerCP-Cy5.5 channel. PI-positive events were excluded prior to analysis.

2.2 Supplementary Table S2 (Excel): TaqMan assays for the four-gene interferon signature (4GS) and reference gene

TaqMan assay IDs (gene symbol, assay ID, catalog number) used to quantify IFI27, IFI44, IFI44L, RSAD2 and the endogenous control 18S rRNA.

2.3 Supplementary Table S3 (Excel): Numerical estimates for within-patient changes in immune-cell parameters

Back-transformed fold changes (month 6 / baseline) with 95% CIs, raw and FDR-adjusted *P* values. Worksheets: README, All, Responders, Non-responders.

2.4 Supplementary Table S4 (Excel): ROC analysis of candidate biomarkers using the modified responder definition (sensitivity analysis)

Data are presented as areas under the receiver operating characteristic (ROC) curve (AUCs) with 95% confidence intervals (CIs). P values were obtained from paired DeLong tests versus the 4GS reference. Sensitivity and specificity were calculated at the optimal cutoffs determined by Youden's J index; units for cutoffs are shown in parentheses. Tph, peripheral helper T cells; 4GS, 4-gene type I IFN signature; MFI, median fluorescence intensity.

2.5 Supplementary Table S5 (Excel): ROC analysis of individual interferon-stimulated gene (ISG) transcripts (sensitivity analysis)

Data are presented as areas under the receiver operating characteristic (ROC) curve (AUCs) with 95% confidence intervals (CIs). ISG, interferon-stimulated gene; 4GS, 4-gene type I IFN signature.

3 Supplementary Method (for Supplementary Figure S5)

The following methods describe the reanalysis of the public SLE PBMC scRNA-seq dataset used for Supplementary Figure S5.

3.1 Dataset and subsampling

GSE135779 (39 SLE, 17 healthy controls) PBMC scRNA-seq data were reanalyzed. Up to 1,000 cells per sample were randomly subsampled (all cells were used when <1,000) and merged for downstream analyses.

3.2 Processing, integration, and annotation

Data were processed in Seurat with LogNormalize (scale factor 10,000), variable-gene selection (~1,200), scaling with regression of nFeature_RNA and percent mitochondrial reads, and PCA, followed by Harmony integration. Harmony-corrected dimensions were used for UMAP visualization and clustering. Clusters were annotated using canonical marker genes and collapsed into macro lineages (B, T, and monocytes; others shown in gray). Erythroid cells were excluded from visualization only.

3.3 4GS and statistics

A scRNA-seq–based 4GS was computed per sample using IFI27/IFI44/IFI44L/RSAD2 as log_2-relative expression versus the healthy-control mean (arithmetic mean across the four genes). For each sample and macro lineage, BST2 was summarized as the median log-normalized expression (≥20 cells/lineage). Spearman correlations between lineage-level BST2 and 4GS or SLEDAI were computed with Fisher's z 95% CIs and BH-adjusted P values.
